# An investigation into the stress-relieving and pharmacological actions of an ashwagandha (*Withania somnifera*) extract: A randomized, double-blind, placebo-controlled study

**DOI:** 10.1097/MD.0000000000017186

**Published:** 2019-09-13

**Authors:** Adrian L. Lopresti, Stephen J. Smith, Hakeemudin Malvi, Rahul Kodgule

**Affiliations:** a College of Science, Health, Engineering, and Education (SHEE), Murdoch University, Perth; b Clinical Research Australia, Duncraig, Western Australia, Australia; c Heamatology Centre Bhopal, Bhopal, Madhya Pradesh; d Saibaba Healthcare, Wagholi, Pune, Maharashtra, India.

**Keywords:** anxiety, ashwagandha, cortisol, stress, testosterone, withania somnifera

## Abstract

**Background:**

Ashwagandha (*Withania somnifera* (L.) *Dunal*) is a herb traditionally used to reduce stress and enhance wellbeing. The aim of this study was to investigate its anxiolytic effects on adults with self-reported high stress and to examine potential mechanisms associated with its therapeutic effects.

**Methods:**

In this 60-day, randomized, double-blind, placebo-controlled study the stress-relieving and pharmacological activity of an ashwagandha extract was investigated in stressed, healthy adults. Sixty adults were randomly allocated to take either a placebo or 240 mg of a standardized ashwagandha extract (Shoden) once daily. Outcomes were measured using the Hamilton Anxiety Rating Scale (HAM-A), Depression, Anxiety, and Stress Scale -21 (DASS-21), and hormonal changes in cortisol, dehydroepiandrosterone-sulphate (DHEA-S), and testosterone.

**Results:**

All participants completed the trial with no adverse events reported. In comparison with the placebo, ashwagandha supplementation was associated with a statistically significant reduction in the HAM-A (*P* = .040) and a near-significant reduction in the DASS-21 (*P* = .096). Ashwagandha intake was also associated with greater reductions in morning cortisol (*P* < .001), and DHEA-S (*P* = .004) compared with the placebo. Testosterone levels increased in males (*P* = .038) but not females (*P* = .989) over time, although this change was not statistically significant compared with the placebo (*P* = .158).

**Conclusions:**

These findings suggest that ashwagandha's stress-relieving effects may occur via its moderating effect on the hypothalamus-pituitary-adrenal axis. However, further investigation utilizing larger sample sizes, diverse clinical and cultural populations, and varying treatment dosages are needed to substantiate these findings.

**Trial registration:**

Clinical Trials Registry—India (CTRI registration number: CTRI/2017/08/009449; date of registration 22/08/2017)

## 1 Introduction

Interest in herbal medicinal products and supplements is high as it is estimated that at least 80% of people worldwide use them for some part of their primary healthcare.^[1]^ Although most ingredients have a long history of traditional use, efficacy has not been clearly established in clinical trials for a large portion of them. Safety also remains uncertain, particularly as cultivation and extraction methods can vary widely. Robustly designed clinical trials are therefore imperative to establish safety and efficacy.

Ashwagandha (*Withania somnifera* (L.) *Dunal*) is a small shrub belonging to the Solanaceae family. It is prolifically grown in dry regions of South Asia, Central Asia, and Africa, and is regularly used in Ayurveda, an ancient Hindu system of medicine.^[2]^ Ashwagandha has been traditionally used to promote “youthful vigour” by enhancing muscle strength, endurance, and overall health.^[3]^ Over 50 chemical constituents have been identified in the various parts of the ashwagandha plant with the major chemical constituents including steroidal alkaloids and lactones, collectively known as withanolides.^[4]^

Pharmacological studies have confirmed that plant preparation of ashwagandha has anti-inflammatory, antioxidant, anticancer, anxiolytic, and immunomodulatory effects. It has also been shown to influence neurological, endocrine, and cardiovascular activity.^[3,5]^ In animal stress models, ashwagandha has been shown to possess anxiolytic, antidepressant, and neuroprotective effects.^[6–8]^ Moreover, animal studies have also confirmed ashwagandha's influence on sex hormone production, as demonstrated by its effects on luteinizing hormone, follicle-stimulating hormone, testosterone, and progesterone.^[9–11]^

Stress is a general term defined as the nonspecific response of the body to any demand for change.^[12]^ It is a major exacerbating factor for both hypertension and diabetes which are major killers worldwide.^[13–15]^ High stress is also a common trigger for mood disorders such as anxiety and depression.^[16,17]^ As an agent to modulate stress and anxiety, ashwagandha has been investigated in several human trials. In 2, double-blind, placebo-controlled studies, ashwagandha was associated with greater reductions in anxiety in adults presenting with predominately generalized anxiety disorder.^[18,19]^ In an 8-week, randomized, double-blind, placebo-controlled study ashwagandha was associated with greater reductions in anxiety, morning cortisol, c-reactive protein, pulse rate, and blood pressure in chronically stressed adults.^[20]^ Greater increases in serum dehydroepiandrosterone sulfate (DHEA-S) and hemoglobin were also noted. Further randomized, double-blind, placebo-controlled studies have confirmed ashwagandha's anti-stress and cortisol-lowering effects in adults with self-reported chronic stress ^[21]^ and chronically stressed overweight and obese adults.^[22]^ In all these studies ashwagandha was well tolerated with minimal adverse effects reported.

These positive trials investigating the anxiolytic and mood-enhancing effects of ashwagandha in adults provide increasing support for the efficacy of this herbal agent for adults suffering from stress and anxiety. Outcomes used to measure efficacy included self-report and clinician-rated instruments, along with measures of physiological markers that are commonly associated with stress and anxiety, including cortisol, pulse rate, and blood pressure. However, the strength of findings is hampered by the small sample sizes used and short treatment duration with no study greater than 8 weeks. In these studies, divergent ashwagandha extracts with varying treatment doses were also used.

The aim of this study was to add to the current body of evidence by investigating the antistress effects and safety profile of a standardized ashwagandha root extract (Shoden) in healthy adults suffering from mild stress. In this 60-day, randomized, double-blind, placebo-controlled study we were also interested in identifying additional mechanisms of ashwagandha's antistress, and mood-enhancing effects, particularly in relation to its influence on steroidal hormones. Accordingly, we examined changes in morning cortisol, DHEA-S, and testosterone levels. We hypothesized that ashwagandha would result in greater reductions in stress and anxiety, serum cortisol, and DHEA-S. As there have been some studies confirming the positive effects of ashwagandha on testosterone, we also predicted greater elevations in serum testosterone levels over time, compared with the placebo.^[23,24]^

## 2 Methods

### 2.1 Study design

**Figure 1:**
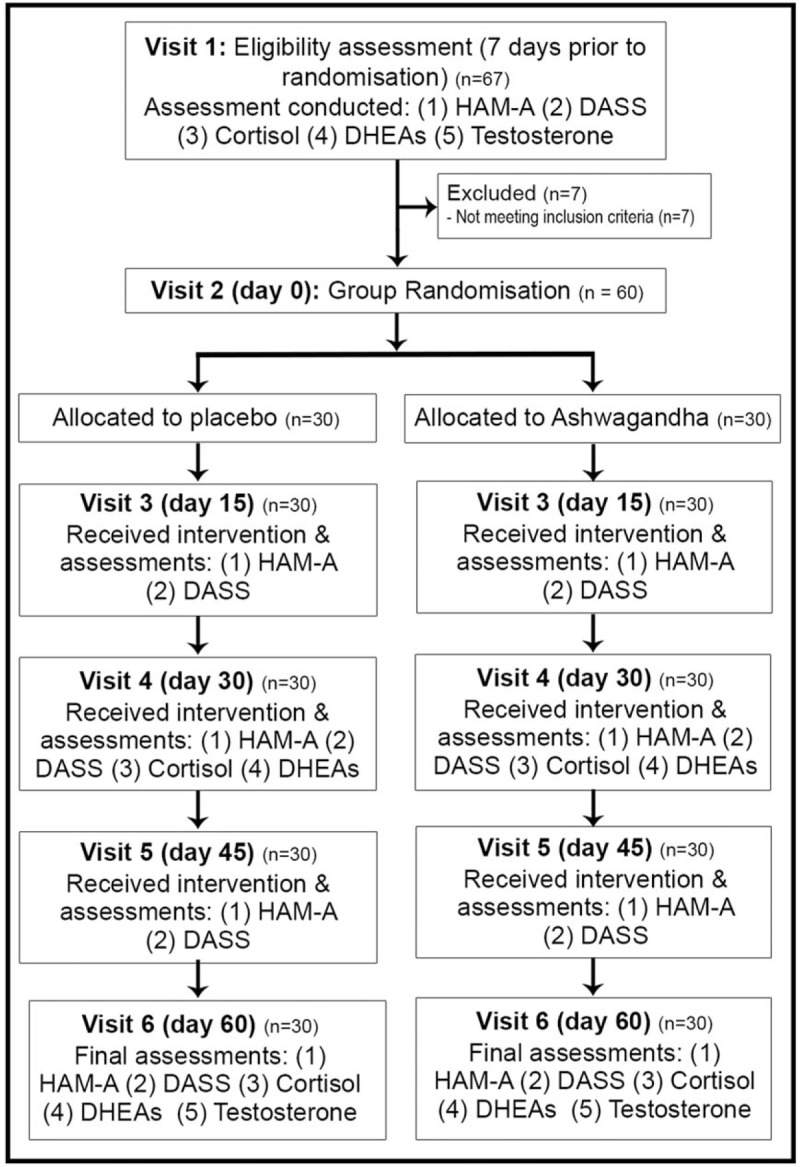
Systematic illustration of study design.

This study was a 60-day, randomized, double-blind, placebo-controlled trial evaluating the efficacy and tolerability of an ashwagandha extract on stress, anxiety, and hormone production in healthy adults. The study protocol was approved by Nagpur Independent Ethics Committee and retrospectively registered with the Clinical Trials Registry- India (CTRI registration number: CTRI/2017/08/009449; date of registration 22/08/2017) with participant recruitment occurring between April 2016 and July 2016. The methods were carried out in accordance with the relevant guidelines and regulations. Details of the study design are outlined in Fig. 1.

An a priori power analysis was undertaken to estimate the required sample size. We predicted a Cohen's d effect size of 0.8 for the treatment group. Assuming a power of 80%, a type 1 error rate (alpha) of 5%, and a 10% drop-out rate, the total number of participants to find an effect was estimated as 57. Enrolled participants were assigned to either 1 of the 2 study groups (ashwagandha or placebo) using a random number table. A randomization list with only the randomization numbers was provided to the study site for the purpose of enrolling volunteers in the study. The master randomization list with the details of allocation was kept safely and confidentially with the study sponsor.

Potential participants were screened after completing a signed informed consent and 60 eligible participants were enrolled in the study as per inclusion and exclusion criteria. Participants attended on 6 occasions to 1 of 2 healthcare centers located in India (Haematology Centre in Bhopal or Sai Baba Healthcare in Pune). During visit 1 (initial assessment conducted approximately 7 days prior to commencement of capsule intake) the following information was collected, or examinations conducted: informed consent, demographic data, medical history, physical, and systemic examination (vital parameters, respiratory rate, electrocardiography, chest x-ray, hematology, biochemistry, and serology), and in women of child-bearing age a pregnancy test was undertaken. Baseline outcome measures were also collected including a morning blood sample (to assess DHEA-S, cortisol, and testosterone), clinician-administered Hamilton Anxiety Rating Scale (HAM-A), and self-report Depression, Anxiety, Stress Scale-21 (DASS-21).

On visit 2 (baseline/day 0) eligible participants meeting criteria eligibility were randomized into 1 of 2 treatment conditions (ashwagandha or placebo). Participants were provided with a 15-day supply of capsules. At visits 3 (day 15), 4 (day 30), 5 (day 45), and 6 (day 60), participants returned to the center, and the following was undertaken: vitals and a physical examination, count of returned pills, provision of new pills, rating administrations (HAM-A and DASS-21), and record of any adverse events. At visit 4 (day 30) and visit 6 (day 60) a morning blood sample was also collected to assess for cortisol and DHEA-S. Assessment of testosterone levels was only undertaken at baseline and day 60.

### 2.2 Participants

Volunteers were obtained from the general population who visited 1 of 2 healthcare centers in India (Haematology Centre in Bhopal or Sai Baba Healthcare in Pune) for a routine health check. Participants were informed about the study, and if agreeable, were assessed by the principal investigator for eligibility based on the following inclusion/exclusion criteria:

#### 2.2.1 Inclusion criteria

Healthy male and female adults aged between 18 and 65 years with a HAM-A between 6 and 17 were eligible to participate. Participants were also willing to participate in the study and comply with its procedures by signing a written informed consent. Female participants of child-bearing age were required to be using a suitable and effective contraceptive method throughout the study and tested negatively on a pregnancy screen. Non-child-bearing women were postmenopausal for at least 12 consecutive months or had undergone surgical sterilization. All participants were encouraged to not make any major lifestyle changes during the study period. They were informed that any major changes may result in exclusion from the study.

#### 2.2.2 Exclusion criteria

Participants were ineligible for participation in the study if they were pregnant, lactating, or were not using an appropriate method of birth control. People with a known hypersensitivity to ashwagandha were also excluded. Individuals with acute narrow-angle glaucoma, prostate hypertrophy, cardiovascular, endocrine or renal disease, or another chronic disease that could affect stress/anxiety or restrict normal, daily function were also ineligible to participate in the study. Individuals who currently, or in the past 6 months, suffered from any diagnosable mental-health disorder (as assessed by the Mini International Neuropsychiatric Interview 6.0) or were taking a psychotropic medication or other herbal preparation were also excluded from participating in the study. People with reported alcohol dependence or were taking any other investigational drug for another clinical trial/research were also ineligible.

### 2.3 Interventions

Capsules containing either 240 mg of an ashwagandha extract (Shoden) or placebo (roasted rice powder) manufactured by Arjuna Natural Ltd, Aluva, Kerala, India were used for the intervention and placebo groups respectively. The dried plant material was visually identified by a qualified botanist as *Withania somnifera* (L.) Dunal and was purchased from a commercial supplier (Neemuch, Madhya Pradesh, India). The extraction solvent used was ethanol:water at a proportion of 70:30 and the extract was standardized by high-performance liquid chromatography to contain 35% withanolide glycosides. Participants were instructed to take 1 capsule (with 84 mg withanolide glycosides), once daily after dinner with 250 mL of water. Capsules were identical in appearance, shape, color, and packaging, comprising oblong green-colored capsules.

### 2.4 Outcome measures

#### 2.4.1 Primary outcome measure 1: Hamilton Anxiety Rating Scale (HAM-A)

The HAM-A is a widely used, well-validated tool consisting of 14 items designed to assess the severity of a patient's anxiety.^[25]^ In this clinician-rated measure, each of the 14 items is rated on a 5-point scale, ranging from 0 = not present to 4 = severe. The HAM-A was completed at initial assessment (7 days prior to capsule administration) and 15, 30, 45, and 60 days after commencement of capsule intake.

#### 2.4.2 Primary outcome 2: Depression, Anxiety, Stress Scale-21 (DASS-21)

The DASS-21 is a validated self-report measure assessing symptoms of stress, anxiety, and depression.^[26]^ Twenty-one questions are rated on a 4-point scale (0–3), ranging from never to almost always (lower scores indicate a reduction in symptoms). The DASS-21 was completed at initial assessment (7 days prior to capsule administration) and 15, 30, 45, and 60 days after commencement of capsule intake.

#### 2.4.3 Secondary outcome measures 1 to 3: serum cortisol, DHEA-S, testosterone

A morning, fasting (approximately 8 am), venepuncture blood sample was collected from participants at the 2 site locations. Levels of serum cortisol and testosterone were measured with the ADVIAÒ Centaur System using competitive immunoassay direct chemiluminescent technology. The IMMULITE 2000 Systems Analyzer was used for the quantitative measurement of DHEA-SO4 in serum. Cortisol and DHEA-S levels were measured at initial assessment (7 days prior to capsule administration), 30 and 60 days after commencement of capsule intake. Testosterone was measured at initial assessment and 60 days after commencement of capsule intake.

#### 2.4.4 Safety assessments

Hematological assessments were undertaken at initial assessment (7 days prior to capsule administration) and 60 days after commencement of capsule intake. These comprised the following: complete blood count (red blood cell, white blood cell, hemoglobin, hematocrit, platelets, and erythrocyte sedimentation rate) and a lipid profile (low-density lipoprotein, cholesterol, high-density lipoprotein, triglycerides, and very-low-density lipoprotein).

### 2.5 Statistical analysis

An independent samples *t* test was used to compare demographic variables across the 2 treatment groups for continuous variables, and Pearson *χ*^2^ was used to compare categorical data. Total scores on the HAM-A, DASS-21 (days 0, 15, 30, 45, and 60), and cortisol, DHEA-S (days 0, 30, and 60) were analyzed for time and treatment (ashwagandha and placebo) effects using a mixed repeated-measures analysis of variance (ANOVA). A paired-samples *t* test was used to analyze changes in testosterone (days 0 and 60) over time. Eta-squared (η^2^) was calculated to examine effect sizes.

There were no significant outliers in data as assessed by the visual inspection of Q-Q plots. The Shapiro–Wilk normality test was conducted to examine the normality of group data. This demonstrated that hormonal data were not normally distributed, and transformations were unable to normalize data. However, a repeated-measures ANOVA was considered the most appropriate option for statistical analyses as it is relatively robust to violations of normality.^[27]^ For all the tests, statistical significance was set at *P* < .05 (2-tailed). All data were analyzed using SPSS (version 24; IBM, Armonk, NY).

## 3 Results

### 3.1 Demographic details and baseline data

A total of 60 participants (37 males and 23 females) were enrolled in the study with all volunteers completing the 60-day trial. Data was also collected in full, with no missing data from assessment instruments. Demographic characteristics are shown in Table 1 and indicate that the study population was homogeneous, with no statistically significant differences between the groups on demographic characteristics.

**Table 1:**
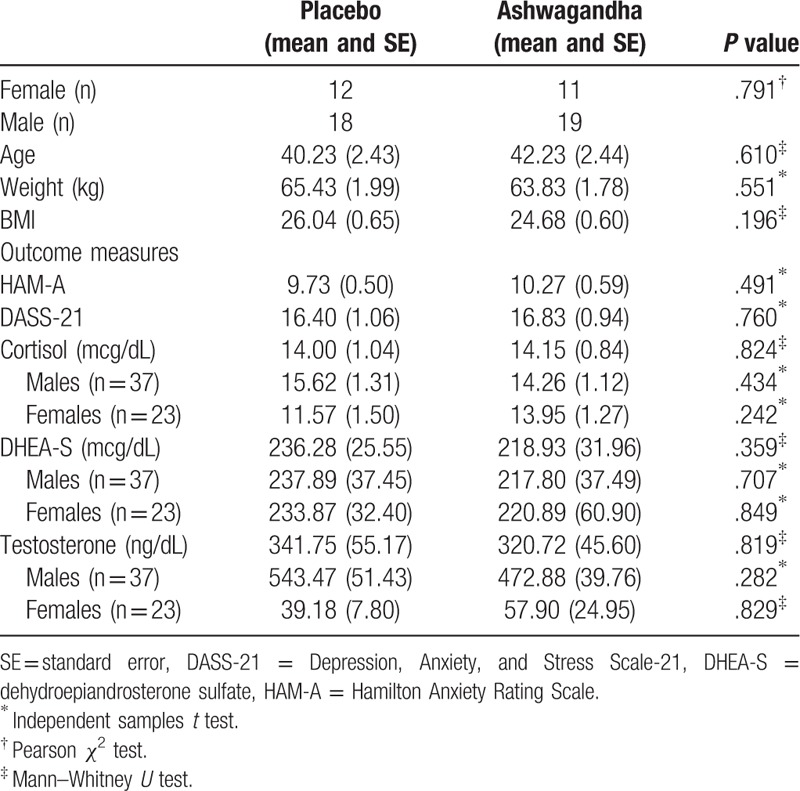
Participant baseline demographic characteristics.

#### 3.1.1 Primary outcome measure 1: HAM-A

**Figure 2:**
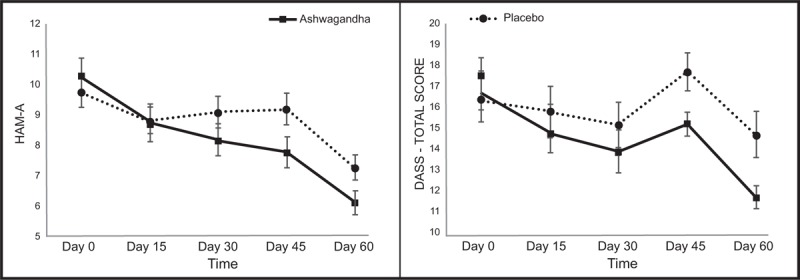
Mean change in mood scores over time (vertical bars depict standard error bars).

Changes in HAM-A scores across the 2 treatment groups and repeated measures ANOVA significance levels are detailed in Table 2 and Fig. 2. A statistically significant 41% reduction in HAM-A was observed over time in the ashwagandha group (F_4,116_ = 15.09, *P* < .001) and a 24% reduction in the placebo group (F_4,116_ = 6.37, *P* < .001). A 34.2% variability across time was observed in the ashwagandha group and an 18% variability in the placebo group. A comparison of between-group differences revealed a statistically significant-time x group interaction (F_4,232_ = 2.55, *P* = .040).

**Table 2:**
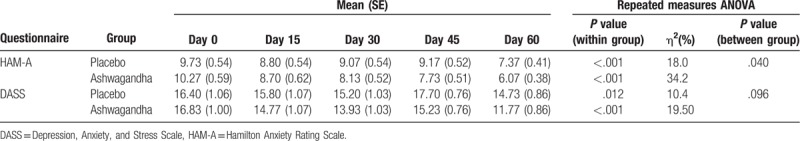
Mood changes during 60-d intervention.

#### 3.1.2 Primary outcome measure 2: DASS-21

Changes in DASS-21 scores across the 2 treatment groups and repeated measures ANOVA significance levels are detailed in Table 2 and Fig. 2. A statistically significant 30% reduction in HAM-A was observed over time in the ashwagandha group (F_4,116_ = 7.03, *P* < .001) and a 10% reduction in the placebo group (F_4,116_ = 3.37, *P* = .012). A 19.5% variability across time was observed in the ashwagandha group and a 10.4% variability in the placebo group. A comparison of between-group differences revealed a near-significant time x group interaction (F_4,232_ = 2.00, *P* = .096).

#### 3.1.3 Secondary outcome measure 1: cortisol

**Figure 3:**
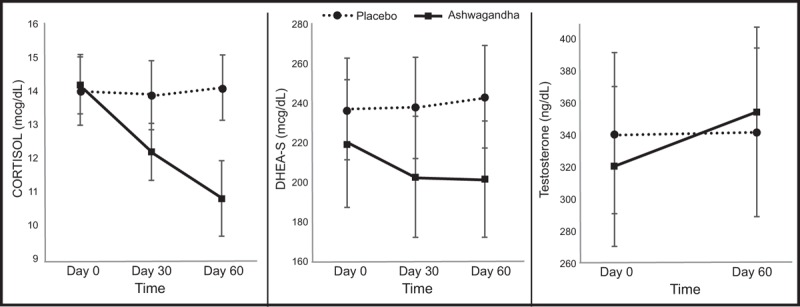
Mean change in hormones (vertical bars depict standard error bars).

Changes in cortisol scores across the 2 treatment groups and repeated measures ANOVA significance levels are detailed in Table 3 and Fig. 3. In the ashwagandha group, a statistically significant 23% reduction in cortisol was observed over time (F_2,58_ = 10.25, *P* =  < .001). However, no significant change occurred in the placebo group (0.5% increase) (F_2,58_ = 0.22, *P* = .800). A 26.1% variability across time was observed in the ashwagandha group and a 0.8% variability in the placebo group. A comparison of between-group differences revealed a statistically significant time x group interaction (F_2,116_ = 8.95, *P* =  < .001).

**Table 3:**
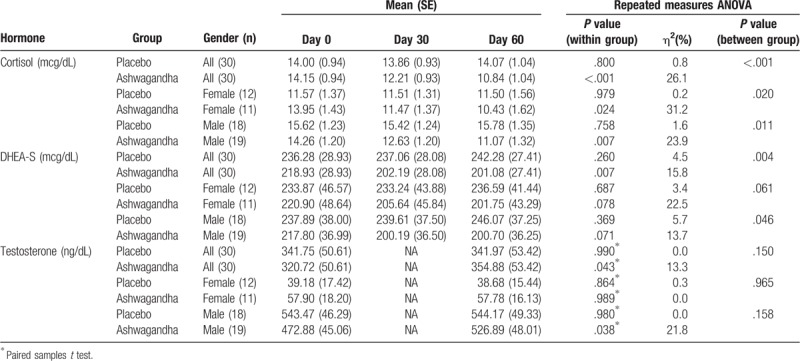
Hormonal changes during 60-d intervention.

Gender-wise comparisons revealed there was a statistically significant 25% reduction in cortisol in females in the ashwagandha group (F_2,20_ = 4.53, *P* = .024) and a nonsignificant 0.6% decrease in the placebo group (F_2,22_ = 0.02, *P* = .979). A 31.2% variability across time was observed in the ashwagandha group and a 0.2% variability in the placebo group. A comparison of between-group differences revealed a statistically significant time x group interaction (F_2,42_ = 4.31, *P* = .020). In males, there was a statistically significant 22% reduction in cortisol in the ashwagandha group (F_2,36_ = 5.64, *P* = .007) and a nonsignificant 1% increase in the placebo group (F_2,34_ = 0.28, *P* = .758). A 23.9% variability across time was observed in the ashwagandha group and a 1.6% variability in the placebo group. A comparison of between-group differences revealed a statistically significant time x group interaction (F_2,70_ = 4.84, *P* = .011).

#### 3.1.4 Secondary outcome measure 2: DHEA-S

Changes in DHEA-S scores across the 2 treatment groups and repeated measures ANOVA significance levels are detailed in Table 3 and Fig. 3. In the ashwagandha group, a statistically significant 8% reduction in DHEA-S was observed over time (F_2,58_ = 5.45, *P* = .007). However, no significant change occurred in the placebo group (2.5% increase) (F_2,58_ = 1.38, *P* = .260). A 15.8% variability across time was observed in the ashwagandha group and a 4.5% variability in the placebo group. A comparison of between-group differences revealed a statistically significant time x group interaction (F_2,116_ = 5.86, *P* = .004).

Gender-wise comparisons revealed a near significant 9% reduction in DHEA-S in females in the ashwagandha group (F_2,20_ = 2.90, *P* = .078) and a nonsignificant 1% increase in the placebo group (F_2,22_ = 0.04, *P* = .687). A 22.5% variability across time was observed in the ashwagandha group and a 3.4% variability in the placebo group. A comparison of between-group differences revealed a near statistically significant time x group interaction (F_2,42_ = 3.00, *P* = .061). In males, there was a near-significant 8% reduction in DHEA-S in the ashwagandha group (F_2,36_ = 2.85, *P* = .071) and a nonsignificant 3% increase in the placebo group (F_2,34_ = 1.02, *P* = .369). A 13.7% variability across time was observed in the ashwagandha group and a 5.7% variability in the placebo group. A comparison of between-group differences revealed a statistically significant time x group interaction (F_2,70_ = 3.21, *P* = .046).

#### 3.1.5 Secondary outcome measure 3: testosterone

Changes in testosterone scores across the 2 treatment groups and repeated measures ANOVA/ paired-samples *t* test significance levels are detailed in Table 3 and Fig. 3. In the ashwagandha group, a statistically significant 11% increase in testosterone was observed over time [T(29) = −2.11, *P* = .043]. However, no significant change occurred in the placebo group (0.1% increase) [T(29) =  −0.01, *P* = .990]. A 13.3% variability across time was observed in the ashwagandha group and a 0% variability in the placebo group. A comparison of between-group differences revealed a nonsignificant time x group interaction (F_1,58_ = 2.13, *P* = .150).

Gender-wise comparisons revealed a nonsignificant 0.2% reduction in testosterone in females in the ashwagandha group [T(10) =  −0.01, *P* = .989] and a nonsignificant 1.3% reduction in the placebo group [T(11) = −0.18, *P* = .864]. A comparison of between-group differences revealed a nonsignificant time x group interaction (F_1,21_ = 0.002, *P* = .965). In males, there was a statistically-significant 11.4% increase in testosterone in the ashwagandha group [T(18) = −2.24, *P* = .038] and a nonsignificant 0.1% increase in the placebo group [T(17) = −0.02, *P* = .980]. A 21.8% variability across time was observed in the ashwagandha group and a 0% variability in the placebo group. A comparison of between-group differences revealed a nonsignificant time x group interaction (F_1,35_ = 2.08, *P* = .158).

### 3.2 Adverse events and treatment compliance

Participants were questioned about capsule tolerability and adverse events at days 15, 30, 45, and 60. Ashwagandha was well tolerated with no significant adverse events reported by participants. Good tolerability of ashwagandha intake was also further confirmed by the ability and willingness of all participants to complete the 60-day trial. Compliance with capsule intake was also high as all participants consumed > 90% of allocated capsules (as measured by returned capsule count at days 15, 30, 45, and 60).

Pre and posthematological measures comprising a full blood count (hemoglobin, white blood cell, neutrophils, eosinophils, platelets, red blood cell, lymphocytes, and monocytes) and lipid profile (total cholesterol, triglycerides, high-density lipoprotein, low-density lipoprotein, and very-low-density lipoprotein) confirmed no statistically significant, between-group differences in the measures over time.

## 4 Discussion

In this randomized, double-blind, placebo-controlled trial, the 60-day intake of an ashwagandha extract (Shoden) in mildly anxious, healthy adults resulted in significant emotional improvements over time. Compared with the placebo, ashwagandha intake was associated with a statistically significant, greater reduction in the HAM-A, although changes in the DASS-21 failed to reach statistical significance, despite a strong positive trend. Ashwagandha intake was also associated with greater reductions in morning cortisol and DHEA-S; and a positive trend suggesting an increase in testosterone concentrations (the latter evidenced in men only). Ashwagandha was well tolerated with no significant reports of adverse events or changes in hematological measures (full blood count and lipid profile) over time.

Based on the HAM-A, anxiety levels reduced by 41% in participants taking ashwagandha, which compared favorably to the 24% reduction experienced in participants taking a placebo. Further confirmation of the mood-enhancing effects of ashwagandha was provided by positive overall improvements in the DASS-21 (30% vs 10%), a measure of depressive, anxiety, and stress symptoms. However, between-group differences in DASS-21 changes did not reach statistical significance. These overall positive anxiolytic and mood-enhancing effects of ashwagandha are consistent with other previously published studies examining the efficacy of other ashwagandha extracts in stressed adults.^[18–22]^ However, the dosage used in this study (240 mg, standardized to be not less than 35% withanolide glycoside) was lower than the 600 mg dose most commonly used in previous studies.

To understand the therapeutic mechanisms of ashwagandha in stressed adults, changes in the stress hormone, cortisol, and steroidal hormones, DHEA-S and testosterone were measured. The findings indicated that compared with placebo, ashwagandha intake was associated with a reduction in fasting, morning cortisol (0.5% increase and 23% reduction, respectively) and DHEA-S (2.5% increase and 8.2% decrease, respectively). Ashwagandha intake was also associated with a statistically significant increase of 10.6%, in testosterone which compared favorably to the 0.1% increase observed in the placebo group. However, changes in testosterone between the ashwagandha and placebo groups did not reach statistical significance.

Gender-wise analyses suggested ashwagandha had similar effects on cortisol and DHEA-S in both males and females as statistically significant changes occurred in both genders. However, testosterone levels did not change significantly in females after both ashwagandha (0.2% reduction) and placebo (1.3% reduction) intake. In contrast, testosterone levels increased in males by 11.4% following ashwagandha intake which compared favorably to the 0.1% increase following placebo intake. However, this difference was not statistically significant, possibly due to the small sample size of 37 males and/or differences in baseline testosterone levels between the treatment conditions. Further investigation utilizing a larger sample size will be required to clarify the significance of these testosterone findings in men. Moreover, the clinical relevance of these changes in testosterone requires further investigation. However, it is helpful to consider that on average it has been reported that testosterone levels reduce by 110 ng/dL every 10 years in males after the age of 30.^[28,29]^ The increase of 54 ng/dL occurring after the 8-week administration of ashwagandha in our study suggests that these changes may have health-related clinical benefits.

Based on our findings, the anxiolytic effects of ashwagandha may be attributed to several mechanisms. First, ashwagandha may have an attenuating effect on the hypothalamic-pituitary-adrenal (HPA) axis activity. In response to a stressor, the HPA axis is associated with a series of responses ultimately leading to increases in both cortisol ^[30]^ and DHEA ^[31,32]^ concentrations. While increased cortisol output has been commonly investigated in human-stress studies, the impact of stress on DHEA has received far less attention. Although higher DHEA is often associated with increased health and longevity,^[33,34]^ within the context of stress its elevation may be an indicator of an increased stress response (or HPA activity). For example, increased DHEA-S secretion has been demonstrated in adults following acute stress exposure,^[31,32]^ and is higher in adults with posttraumatic stress disorder, as confirmed by a recent meta-analysis.^[35]^ Higher levels are also associated with cigarette smoking and alcohol consumption in middle-age men.^[36]^ These findings suggest elevated DHEA (along with cortisol) may be a marker of increased stress. Its acute reduction may, therefore, be a sign of stress reduction. Within the adrenal cortex, production of cortisol and DHEA occurs in different layers, with cortisol produced in the zona fasciculata and DHEA in the zona reticularis. Although a regulatory negative feedback system is in place to ensure a restoration in cortisol levels following a stressor, a significant body of evidence suggests that anxiety and depressive disorders are associated with disturbances in HPA axis activity, commonly leading to an excess in cortisol secretion.^[37]^ The reduction in morning cortisol and DHEA-S in participants taking ashwagandha suggest it has a moderating effect on HPA axis activity in stressed adults. This may be associated with stress-lowering effects as the stress response (or HPA axis activity) becomes less reactive to stressors.

Although not investigated in this study, other potential mechanisms of ashwagandha's anxiolytic effects may be via it antioxidant and anti-inflammatory effects.^[38,39]^ Inflammation and oxidative stress are increased during times of high stress, and higher levels have been demonstrated in adults with depression and anxiety.^[40,41]^ In preclinical studies, ashwagandha can also influence GABAergic ^[42]^ and serotonin activity,^[8,43]^ which have antidepressant and anxiolytic effects. Moreover, despite these mechanisms being discussed separately, their effects do not occur in isolation and it is likely that the interaction of all these mechanisms may be responsible for the positive, mood-enhancing effects of ashwagandha.

### 4.1 Study limitation and directions for future research

Although findings from this study add to the body of evidence supporting the antistress effects of Ashwagandha, there remain several unanswered questions. In this study, healthy adults with mild stress were recruited. The effects of ashwagandha in clinical populations suffering from an anxiety or other affective disorder remain to be determined. However, positive anxiolytic effects of ashwagandha in adults with generalized anxiety disorder have been identified in previous studies.^[18,19]^ Moreover, most of the studies on the anxiolytic effects of ashwagandha, including ours, have been conducted in India. Future studies with other cultural populations may, therefore, be useful. We also did not examine the impact of feeding habits, economic conditions, and daily occupation on the anti-stress effects of ashwagandha. These may have an influence on therapeutic outcomes so would be worth examining in future studies.

In this study, the effects of ashwagandha were examined over a 60-day duration, which is consistent with most studies. Longer studies should be undertaken to examine the safety and efficacy of ashwagandha supplementation over a longer period. Follow-up after intake cessation will also be helpful to identify if there are any withdrawal issues and whether positive changes are sustained over time once supplementation is ceased.

In the 6 studies that have now examined the anxiolytic effects of ashwagandha, varying ashwagandha extracts, using different extraction techniques and standardization methods, have been used. Overall, the findings have been positive; however, the relative safety and anxiolytic potency of these different extracts are yet to be examined. This may be of interest in future trials. Moreover, dosages used across trials have varied significantly making it difficult to decipher optimal therapeutic doses. Dose-escalation studies for nonresponders and dose-response effects may also be of interest. In a study by Auddy et al, ^[20]^ greater dose-response effects were identified in chronically stressed adults.

Finally, we examined the physiological impact of ashwagandha supplementation in stressed adults and identified several changes in hormones associated with the adrenal and steroidal system. Given the small sample size recruited in our study, we were unable to examine the relevance of such changes for symptom resolution. In future trials, it will not only be important to examine physiological changes associated with ashwagandha supplementation, but also to determine whether such changes are related to symptomatic changes. This will help us develop clearer hypotheses about ashwagandha's anxiolytic mechanisms of action. Moreover, further investigation into the effects of ashwagandha on other hormones such as pregnenolone, oestrogens, and progesterone; and examinations into gender differences will be useful.

In conclusion, the findings from this study support the positive anxiolytic effects of a novel ashwagandha extract (Shoden, standardized to not less than 35% of total glycowithanolides) taken for 60 days at a dose of 240 mg daily. Statistically significant, between-group differences were confirmed by 1 mood measure (HAM-A), while a strong positive trend was observed for the DASS-21 measure. The ashwagandha extract was well-tolerated with no reported significant adverse effects. Supplementation was associated with a reduction in cortisol and DHEA-S, and a positive, although nonsignificant trend of increased testosterone in men. These results suggest the anxiolytic effects of ashwagandha in stressed adults may be associated with an attenuating effect on HPA axis activity and, in men, increased testosterone production. However, there are potentially additional mechanisms of action that were not investigated in our study. Further clinical trials are warranted using larger sample sizes, differing cultural populations, and longer durations to substantiate our findings and those from previously conducted trials.

## Acknowledgment

The authors gratefully acknowledge Arjuna Natural Ltd for funding the project and supplying Shoden for use in this study.

## Author contributions

**Conceptualization:** Hakeemudin Malvi, Rahul Kodgule.

**Data curation:** Rahul Kodgule.

**Formal analysis:** Adrian Lopresti, Stephen J Smith.

**Funding acquisition:** Rahul Kodgule.

**Investigation:** Hakeemudin Malvi, Rahul Kodgule.

**Methodology:** Adrian Lopresti, Stephen J Smith, Hakeemudin Malvi, Rahul Kodgule.

**Project administration:** Hakeemudin Malvi, Rahul Kodgule.

**Writing – original draft:** Adrian Lopresti, Stephen J Smith.

**Writing – review & editing:** Adrian Lopresti, Stephen J Smith, Hakeemudin Malvi, Rahul Kodgule.

Adrian Lopresti orcid: 0000-0002-6409-7839.
